# The Effects of Temperature on Dynamics of Psychiatric Outpatients

**DOI:** 10.3389/fpsyt.2020.523059

**Published:** 2020-12-07

**Authors:** Ying Shao, Jiahui Xu, Ying Qiao, Yang Shao, Jian-Ming Fei

**Affiliations:** ^1^Hospital Administration Office, Shanghai Mental Health Center, Shanghai Jiao Tong University School of Medicine, Shanghai, China; ^2^Department of Biostatistics, St. Jude Children's Research Hospital, Memphis, TN, United States; ^3^Shanghai Mental Health Center, Shanghai Jiao Tong University School of Medicine, Shanghai, China

**Keywords:** season, temperature, psychiatry, outpatients, mood

## Abstract

**Background:** Climate changes affect mental states and alter the risk for psychiatric diseases. Seasonal changes in temperature lead to dynamics in the occurrence of psychiatric conditions and pose challenges in the administration of clinical psychiatry services.

**Methods:** The present study aims to retrospectively analyze outpatient data with weather reports from January 2014 to March 2019 at Shanghai Mental Health Center, one of the largest psychiatric hospitals in the world, in order to provide policy insights into the administration of psychiatric clinics.

**Results:** The results show steady increases in the number of overall patients over the past 5 years with several peaks within each year. Temperature changes and weather information reliably predict the increased number of psychiatric patients.

**Conclusions:** We conclude that mental health hospitals should prepare for patient dynamics based on the weather forecast.

## Background

Environmental changes serve as important risk factors for health. Mental disorders are associated with different aspects of environmental conditions, such as average outdoor temperature, air quality, temperature variation, and seasonal amount of daily sunlight (1–3). These conditions can either directly induce psychosocial stress within individuals or generate confounding effects on physical inactivity, lifestyle, hormonal changes, and social interactions, which then result in changes in mental states (4, 5). Previous studies show that daily extreme weather conditions accompany increased risk for psychiatric disorders (6). It is also found that air pollution and temperature variability lead to declined mental health conditions (7–9). It is unclear how seasonal mild temperature variation is associated with the occurrence or recurrence of mental diseases.

Recent studies employing time-series analyses highlight the potential predictive effects of ambient temperature on mental health conditions (1). For instance, extreme temperatures increase the hospital admission of patients with mental disorders (1, 10), schizophrenia (11) and emergency mental disorders (12). However, few studies have systemically examined the association between temperature and psychiatric hospital outpatient visits over a prolonged period.

In practical management of clinical psychiatric services, it is important to predict the number of outpatients in the coming season. Located in Shanghai, China, a city without extreme weather conditions, the Shanghai Mental Health Center is one of the largest psychiatric clinical service centers in the world with nearly 1 million outpatient visits annually. We, here, retrospectively analyze the dynamics of outpatients visiting the center from January 2014 to March 2019 together with the temperature changes during this period. The results suggest an effect of environmental–cultural interaction on psychiatric outpatients.

## Methods

### Study Design and Participants

The summary of outpatient information was extracted from a database for the period from January 2014 to March 2019. The summary information for total outpatients and outpatients for the general psychiatric services (first time or recurring), psycho-consultation (first time or recurring), and children's psychiatric services (first time or recurring) subdivisions/departments were obtained as outcome variables.

The weather information with monthly average temperatures was obtained from a Shanghai weather report website, retrospectively. The monthly highest and lowest temperatures were taken as predictive variables for analyses.

### Statistical Analyses

Variance and Spearman's correlation coefficient were calculated to explore the direction, magnitude, and variation of temperature and outpatient volume center-wide or in the subdivisions as well as their relationship.

All statistical analyses were performed using R 3.5.3. *P* < 0.05 was used as the significance level.

## Results

### General Trend

**Figure 1:**
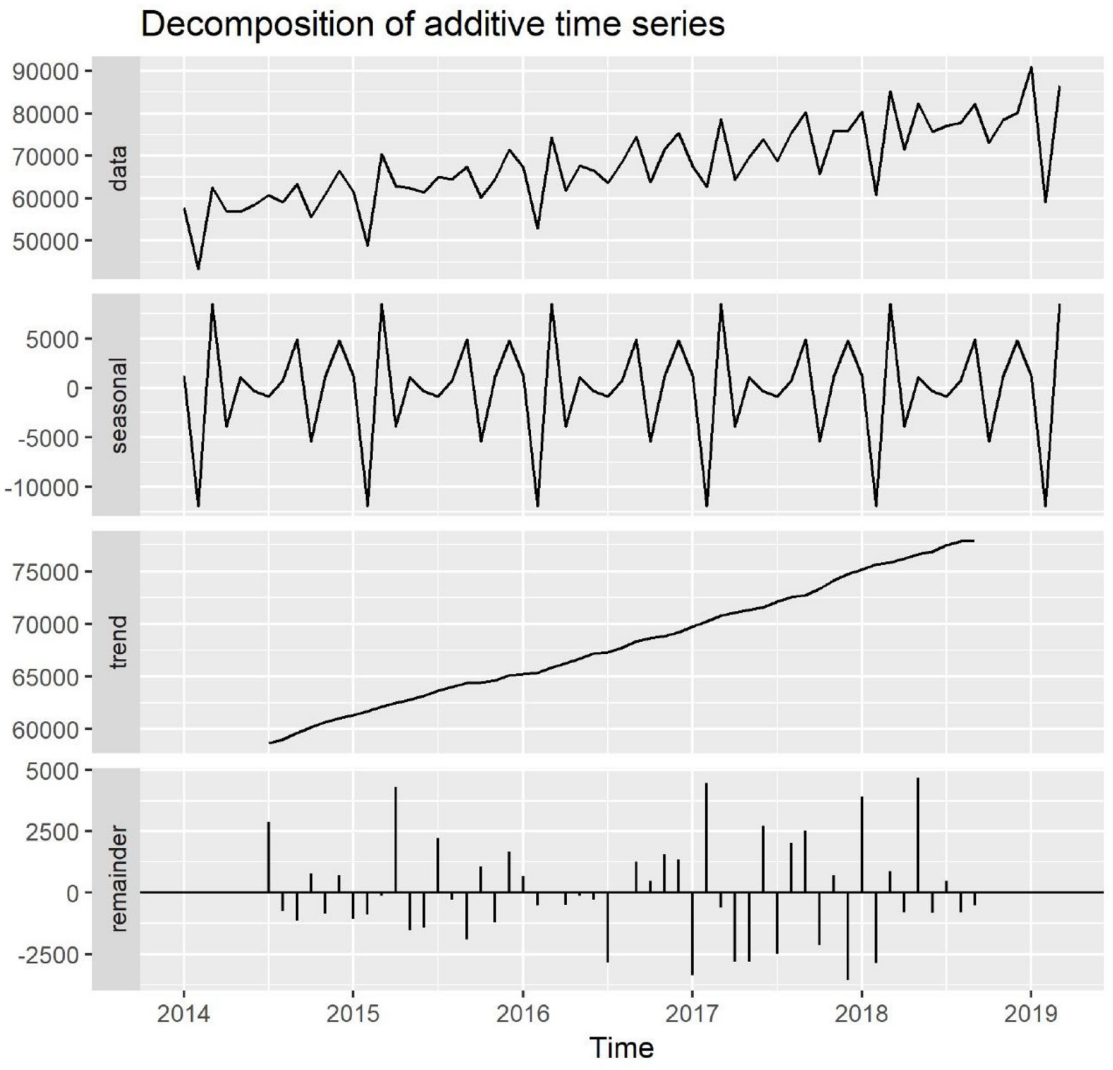
General and seasonal trends of total outpatients at the Shanghai Mental Health Center from January 2014 to March 2019.

**Figure 2:**
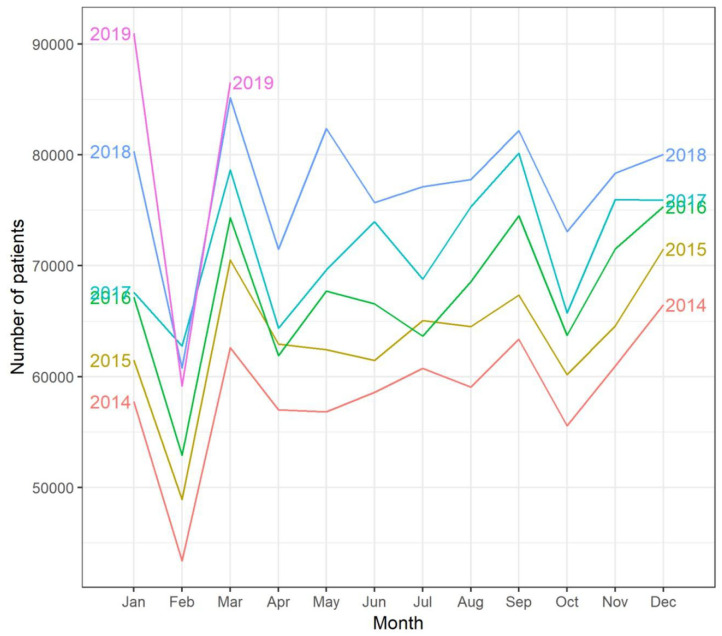
Seasonal plot of total outpatients at the Shanghai Mental Health Center from January 2014 to March 2019.

We observed a steady increase in total outpatients per month during the past 5 years (2014–2018). Seasonal variation patterns for total outpatients were similar across the 5 years with the lowest number occurring in February and the second lowest in October (Figures 1, 2).

The three subdivisions, general psychiatric services, psycho-consultation, and children's psychiatric services, showed similar increasing trends as well as seasonal dynamic patterns to the total outpatients over the past 5 years.

### Effects of Concurrent Monthly Temperature on Outpatients

Based on the correlation analysis, it is suggested that, in the general psychiatry subdivision, both the highest (*r* = 0.282, *P* = 0.025) and lowest (*r* = 0.281, *P* = 0.025) monthly temperatures were significantly positively related to the number of first-time outpatients rather than recurring outpatients.

Similarly, in the psycho-consultation subdivision, both the highest (*r* = 0.421, *P* = 0.001) and lowest (*r* = 0.412, *P* = 0.001) monthly temperatures are suggested to have a significant positive relation with the number of first-time outpatients but not recurring outpatients.

However, neither the highest nor the lowest monthly temperature showed a significant relation with the number of first-time (*P* = 0.061 and 0.050, respectively) or recurring outpatients for the children's psychiatry subdivision (Table 1).

**Table 1:** The effects of average temperature of concurrent and lagged month on number of outpatients (Spearman's correlation analyses, showing *r* and *P*-value).

		**Highest temperature**	**Lowest temperature**
**CONCURRENT MONTH**			
Total outpatients		0.068 *P* = 0.595	0.074 *P* = 0.563
General psychiatry	Total	0.049 *P* = 0.704	0.054 *P* = 0.673
	First-time	0.282 *P* = 0.025 ^*^	0.281 *P* = 0.025^*^
	Recurring	0.018 *P* = 0.892	0.022 *P* = 0.866
Psycho-consultation	Total	0.096 *P* = 0.457	0.102 *P* = 0.425
	First-time	0.421 *P* = 0.001^*^	0.412 *P* = 0.001^*^
	Recurring	0.037 *P* = 0.774	0.045 *P* = 0.725
Children psychiatry	Total	0.03 *P* = 0.813	0.035 *P* = 0.784
	First-time	0.238 *P* = 0.061	0.248 *P* = 0.05
	Recurring	−0.035 *P* = 0.785	−0.035 *P* = 0.786
**LAGGED MONTH**			
Total outpatients	0.144 *P* = 0.261	0.133 *P* = 0.299	0.133 *P* = 0.299
General psychiatry	Total	0.131 *P* = 0.307	0.123 *P* = 0.337
	First-time	0.189 *P* = 0.139	0.179 *P* = 0.161
	Recurring	0.112 *P* = 0.38	0.104 *P* = 0.417
Psycho-consultation	Total	0.146 *P* = 0.253	0.13 *P* = 0.309
	First-time	0.304 *P* = 0.015^*^	0.276 *P* = 0.028^*^
	Recurring	0.110 *P* = 0.391	0.096 *P* = 0.454
Children psychiatry	Total	0.109 *P* = 0.397	0.114 *P* = 0.373
	First-time	0.292 *P* = 0.02^*^	0.291 *P* = 0.021^*^
	Recurring	0.035 *P* = 0.787	0.040 *P* = 0.758

* *P < 0.05*.

### Effects of Lagged Monthly Temperature on Outpatients

We further examined if averaged temperatures from the preceding month were related to outpatient volume. The highest and lowest temperatures with a 1-month lag did not show any significant correlation to the number of first-time and recurring outpatients in the general psychiatry subdivision.

On the other hand, the 1-month lagged highest (*r* = 0.304, *P* = 0.015) and lowest (*r* = 0.276, *P* = 0.028) temperatures were suggested to be significantly positively related to the number of first-time outpatients in the psycho-consultation subdivision but not for recurring outpatients.

Last but not least, 1-month lagged highest (*r* = 0.292, *P* = 0.020) and lowest (*r* = 0.291, *P* = 0.021) temperatures were suggested to have a significant positive relation to the number of first-time outpatients in the children's psychiatry subdivision but not recurring outpatients.

## Discussion

The study demonstrates that temperature factors impact the number of clinical psychiatric outpatients significantly. Higher temperatures can be associated with increased psychiatric conditions as evidenced by the increased number of outpatient visits to public clinics. This makes it possible for public health administration departments to forecast the volume of psychiatric outpatients by taking monthly or even weekly and daily temperature dynamics into consideration.

The concurrent monthly temperature had a stronger effect on first-time rather than recurring patients in all subdivisions (marginally significant for children's psychiatry), suggesting that the current temperature did impact the mood status of already diagnosed patients, but it might act as an important cause for a patient's first episode of mental disruption. Notably, 1-month lagged temperatures were correlated to number of first-time outpatients in psycho-consultation and children's psychiatry, but not in general psychiatry. It is possible that the general psychiatry patients were more sensitive to high temperatures, and they sought treatment at clinics immediately after the temperature change while the psycho-consultation patients had mild conditions and the children were taken to the clinic by their parents, both of which resulted in prolonged treatment-seeking timelines.

It is also notable that the number of recurring outpatients did not show significant correlation to temperature conditions. It is highly possible that these patients were compliance with treatment and some knowledge of their own diseases, and therefore, they could partly self-adjust to environmental changes. However, it is important to divide these patients into different subcategories, such as bipolar disorder, schizophrenia, and major depression, in order to perform detailed analyses for each psychiatric condition.

Notably, for the years included in the analyses, seasonal dynamics were similar across different years although with a yearly increasing trend. February happened to have the fewest outpatients in a year. This is potentially due to the Chinese New Year festival; as a cultural period for family union, patients may have tended to take vacations to travel back home. This suggests that social factors interact with environment changes in prediction of outpatient visits.

## Conclusion

In conclusion, the present study reports that environmental temperature correlates to the number of psychiatric outpatients. This offers some insight for hospital management into improving outpatient service qualities.

## Data Availability Statement

The datasets generated for this study are available on request to the corresponding author.

## Ethics Statement

Ethical review and approval was not required for the study on human participants in accordance with the local legislation and institutional requirements. Written informed consent was not required to participate in this study in accordance with the national legislation and the institutional requirements.

## Author Contributions

YaS and J-MF designed the study. YiS, JX, YQ, YaS, and J-MF performed the study, analyzed the results, and wrote the paper together. All authors have read and approved the final version of the manuscript.

## Conflict of Interest

The authors declare that the research was conducted in the absence of any commercial or financial relationships that could be construed as a potential conflict of interest.
